# Dysregulation of Vesicular Glutamate Transporter VGluT2 *via* BDNF/TrkB Pathway Contributes to Morphine Tolerance in Mice

**DOI:** 10.3389/fphar.2022.861786

**Published:** 2022-04-26

**Authors:** Liqiong He, Wei Xu, Chengliang Zhang, Zhuofeng Ding, Qulian Guo, Wangyuan Zou, Jian Wang

**Affiliations:** ^1^ Department of Anesthesiology, Xiangya Hospital, Central South University, Changsha, China; ^2^ National Clinical Research Center for Geriatric Disorders, Xiangya Hospital, Central South University, Changsha, China; ^3^ Department of Anesthesiology, Hunan Provincial Maternal and Child Health Care Hospital, Changsha, China; ^4^ Department of Cardiovascular Surgery, Xiangya Hospital, Central South University, Changsha, China

**Keywords:** VGluT2, morphine tolerance, brain-derived neurotrophic factor, spinal cord, hyperalgesia

## Abstract

Morphine is widely used in the treatment of moderate to severe pain. Long-term use of morphine leads to various adverse effects, such as tolerance and hyperalgesia. Vesicular glutamate transporter 2 (VGluT2) accumulates glutamate into synaptic vesicles and plays multiple roles in the central nervous system. However, the specific role of VGluT2 in morphine tolerance has not been fully elucidated. Here, we investigated the regulatory role of VGluT2 in morphine tolerance and assessed the potential role of the brain-derived neurotrophic factor (BDNF)/tyrosine kinase B (TrkB) pathway in VGluT2 mediated morphine antinociceptive tolerance in mice. In the present study, we found that VGluT2 is upregulated in the spinal cord after the development of morphine tolerance. Furthermore, inhibition of VGluT2 with its antagonist (Chicago sky blue 6 B, CSB6B) or knockdown of VGluT2 by lentivirus restored the analgesic effect of morphine, suppressed the activation of astrocytes and microglia, and decreased glial-derived pro-inflammatory cytokines. Overexpression of VGluT2 by lentivirus facilitated morphine tolerance and mechanical hyperalgesia. In addition, we found the expression of BDNF is correlated with VGluT2 expression in the spinal cord after chronic morphine administration. Intrathecal injection of the BDNF/TrkB pathway antagonist K252a attenuated the development of morphine tolerance and decreased the expression of VGluT2 in the spinal cord, which suggested the BDNF/TrkB pathway participates in the regulation of VGluT2 in morphine tolerance. This study elucidates the functional capability of VGluT2 in modulating morphine tolerance and identifies a novel mechanism and promising therapeutic target for morphine tolerance.

## 1 Introduction

Morphine is widely used in the treatment of various acute and chronic pain (Bohn et al., 2000; Corder et al., 2017). Long-term use of morphine results in various adverse effects, including constipation, itching, and morphine tolerance (Stein and Machelska, 2011). Morphine tolerance is characterized by a decrease in analgesic efficacy following repeated administration that can be overcome by dose escalation (Harrison et al., 1998; Koshimizu et al., 2018). The development of morphine tolerance is often accompanied by enhanced pain sensitivity such as hyperalgesia (Xu et al., 2014; Kliewer et al., 2019). The mechanisms of morphine tolerance are very complicated, including the desensitization and internalization of opioid receptors (Crain and Shen, 2000; Heinke et al., 2011; Williams et al., 2013), the interaction between various subtypes of opioid receptors (Gomes et al., 2004; Rozenfeld et al., 2007), the neuroinflammatory response caused by glial cells (Ferrini et al., 2013; Cai et al., 2016), and the upregulation of NMDA receptors (Mao, 1999; Zhang et al., 2018). However, the specific mechanism has not been fully understood, and there is a lack of effective treatments in clinical practice.

Glutamate is an important excitatory neurotransmitter implicated in pain modulation, morphine-induced tolerance, and hyperalgesia (Nedergaard et al., 2002; Forde, 2014; Miladinovic et al., 2015). The extracellular homeostasis of glutamate is determined by excitatory glutamate transporters (EAATs), which eliminate glutamate from the synaptic cleft, and vesicular glutamate transporters (VGluTs), which transport glutamate into presynaptic vesicles before exocytotic release. While several lines of studies have shown that EAATs play an important role in morphine tolerance (Mao et al., 2002; Xu et al., 2003; Yang et al., 2008), the specific role of VGluTs in morphine tolerance has not been fully studied. Notably, there are three isoforms in the VGluT family, VGluT1, VGluT2, and VGluT3. VGluT1 is mainly expressed in non-nociceptive neural terminals, and mice heterozygous for VGluT1 display no changes in pain behavior (Leo et al., 2009). VGluT2 is mainly expressed in nociceptive primary afferents, knockdown of VGluT2 in mice showed alleviation in neuropathic pain sensitivity (Moechars et al., 2006; Brumovsky et al., 2007). VGluT3 is expressed in a small group of primary afferents and is associated with mechanical pain sensation (Seal et al., 2009). Given that the nociceptive primary afferents are the main sites of action for the analgesic effects of morphine, we postulated that VGluT2 may play a key role in morphine tolerance.

BDNF, which binds to the TrKB receptor with high specificity and affinity, exerts an important influence in the development of morphine tolerance (Matsushita and Ueda, 2009; Khoshdel et al., 2019). Recent studies have shown that the BDNF/TrkB pathway participates in the modulation of VGluT2 expression in isolated hippocampal neurons (Melo et al., 2013). Moreover, administration of the TrkB antagonist ANA-12 in the nucleus accumbens reduced VGluT2 expression in the hypothalamus and basolateral amygdala (Azogu and Plamondon, 2017). Based on this, we hypothesized that the BDNF/TrkB pathway regulates the expression of VGluT2 in the spinal cord, contributing to the development of morphine tolerance in mice.

Therefore, in the present study, we investigated the regulatory role of VGluT2 in morphine tolerance and explored the potential effect of the BDNF/TrkB pathway on the regulation of VGluT2 in morphine tolerance mice.

## 2 Methods

### 2.1 Animals

Male ICR mice weighing 28–32 g at the beginning of the study were provided from Hunan SJA Laboratory Animal Co., Ltd. All mice were raised in standard transparent plastic cages under a temperature-controlled environment, with a 12 h light/dark cycle, and had ad libitum access to food and water. All experimental procedures and protocols were approved by the Animal Care and Use Committee of Central South University and adhered to the National Institute of Health Guide on the Care and Use of Laboratory Animals. Animals were randomly assigned to groups using a random number table. All animals’ behavioral tests were performed in a double-blinded fashion.

### 2.2 Induction of Morphine Tolerance

The model of morphine tolerance was constructed as described previously (31). Briefly, ICR mice were subcutaneously injected with morphine (Northeast Pharm, China) (10 mg/kg) twice daily (9 a.m. and 6 p.m.) for seven consecutive days. The mice from the control group were subcutaneously injected with the same dose of normal saline.

### 2.3 Behavioral Assessments

#### 2.3.1 Tail-Flick Test

The tail-flick assay was employed to evaluate the antinociception of morphine according to the methods described previously (Huang et al., 2019). Briefly, mice were gently restrained in the fixator, and one-third of the tail from the tip was dipped into a 52 ± 0.2 °C water bath. The latency of the tail removal from the hot water was recorded. The cut-off time was set at 10 s. The tail-flick test was performed 30 min before morphine or saline injection and then repeated 30 min later. The percentage of maximum possible antinociceptive effect (%MPE) was calculated according to the formula: %MPE=(test latency–predrug latency)/(cut-off time–predrug latency)×100% (Hayhurst and Durieux, 2016).

#### 2.3.2 Mechanical Pain Threshold Measurement

To test the mechanical pain threshold, mice were separated and placed on a metal grid with a plastic partition and habituated for 30 min. A series of Von Frey hairs were applied vertically on the hind paw of the mouse. One filament was applied three times with an interval of 5 s. A positive reaction was defined as a quick withdrawal of the hind paw or licking of the paw. The lowest filament evoked positive responses was recorded as the paw withdrawal mechanical threshold. Each test was repeated three times with an interval of 5 min. Von Frey test was performed before morphine or saline injections.

### 2.4 Drugs and Lentivirus Delivery

The vesicular glutamate transporter inhibitor Chicago Sky Blue 6 B (CSB6B) was purchased from Tocris Bioscience (UK). CSB6B was dissolved in 0.9% saline at a dose of 1 μg/μL. On day 7 after morphine administration, 5 μL of CSB6B or 5 μL of saline (vehicle) was intrathecally injected immediately after the morphine infusion. The CSB6B dose was chosen based on previous experiments (Yu et al., 2013). The BDNF/TrkB pathway inhibitor, tyrosine kinase inhibitor K252a (Sigma-Aldrich, United States) was dissolved in dimethyl sulfoxide (DMSO) and diluted in normal saline to a concentration of 0.04 μg/μL (final concentration of DMSO was 0.02%). K252a (0.2 μg/5 μL) was injected intrathecally (i.t.) for seven consecutive days 30 min before the morphine injection. The dose of K252a was selected based on previous experiments (Renn et al., 2011). The vehicle control for K252a was 0.2% DMSO.

The lentiviral vector (pGLV3/H1/GFP) containing VGluT2 targeting shRNA (LV-shVGluT2), the lentiviral vector (pGLV5/EF-1a/GFP) containing plasmids encoding VGluT2 to overexpress VGluT2 (LV-VGluT2), and the scrambled control (LV-NC) were purchased from Genepharma (Genepharma, China). The sequence of the VGluT2 targeting shRNA was designed as previously described with the following sequence: 5′-GCTCACCTCTACCC TCAATAT-3’ (Zhang et al., 2020). The sequence of scrambled control was 5′-TTC​TCC​GAA​CGT​GTC​ACG​T-3′, which showed no homology with any known mammalian gene. The titer of each lentivirus was 1×10^9^ TU/mL. Each lentivirus (7.5 μL) was intrathecally injected, and the efficiency of lentivirus infection was validated by the western blotting test.

Intrathecal injection was performed in awake mice as previously described (Huang et al., 2019). The iliac crest of the mouse was gently gripped, and a 30 gauge needle attached to a 25 μL Hamilton syringe was inserted into the intervertebral space between the L5 and L6 vertebrae, and a reflexive flick of tail indicated the accuracy of each injection. When the reflexive flick of the tail was observed, lentivirus or drugs was slowly delivered in 1 min.

### 2.5 Western Blotting

Animals were sacrificed after deeply anesthetized with isoflurane, total proteins from the lumbar enlargements of spinal cords were extracted by ice-cold Radio Immunoprecipitation Assay (RIPA) lysis. Proteins were separated on SDS-PAGE gel and transferred to polyvinylidene difluoride (PVDF) membrane (Merck Millipore, United States). After blocking with 5% nonfat milk, the samples were incubated with rabbit anti-β-actin (1:3000, Abcam, United States), rabbit anti-GAPDH (1:3000, Merck Millipore, United States), mouse anti-VGluT2 (1:2000, Abcam, United States), goat anti-Iba-1 (1:2000, Wako, Japan), rabbit anti-GFAP (1:2000, Abcam, United States), rabbit anti-BDNF(1:2000, Abcam, United States), or rabbit anti-TrkB (1:2000, Abcam, United States) at 4 °C overnight. The next day, the corresponding rabbit, goat, or mouse Horseradish peroxidase (HRP)-conjugated secondary IgG (1:20000, Jackson ImmunoResearch, United States) was incubated for 1 h at room temperature. The gray value of the protein on the membrane of the corresponding molecular size was detected using western blotting detection kit (WesternBright Sirius, Advansta, United States) and ChemiDoc XRS imaging system (Bio-Rad). Image Lab 3.0 system (Bio-Rad) was utilized to perform standardized analysis on the test results.

### 2.6 Glutamate Concentration Assay

Glutamate concentrations were determined using a Glutamate Colorimetric Assay Kit (K629, BioVision, United States). Glutamate concentrations were measured according to the manufacturer’s instructions. In brief, the lumbar enlargement of the spinal cord was collected from the mice and homogenized in 100 μL of glutamate assay buffer. Then the samples were centrifuged at 13,000 g at 4°C for 10 min, and the supernatants were collected. After the glutamate assay reagents were added and mixed, the reaction was incubated for 30 min at 37°C. A microplate reader (Versa Max, Molecular Devices) was employed to measure the optical density (OD) at 450 nm and glutamate concentrations were calculated.

### 2.7 Quantitative Real-Time PCR (qRT-PCR)

Total RNA was extracted using the TransZol Up (Transgen, China) and cDNA was synthesized using First-Strand cDNA Reverse Transcription SuperMix (Transgen, China). Then, qPCR was performed with SYBR Green qPCR SuperMix (Transgen, China) on the ABI QuantStudio five instrument. Primer sequences are listed in Table 1. All qPCR results were calculated using the 2^−ΔΔCT^ method (Livak and Schmittgen, 2001).

**TABLE 1:** The detailed information of primer sequence.

Sequence Name	Primer Sequence
β-actin	F:5′-CATCCTGCGTCTGGAACCTGG-3′
	R: 5′-TAA​TGT​CAC​GCA​CGA​TTT​CC-3′
IL-6	F: 5′-AGT​TGC​CTT​CTT​GGG​ACT​GAT​GTT​G-3′
	R: 5′-GGT​ATC​CTC​TGT​GAA​GTC​TCC​TCT​CC-3′
IL-1β	F: 5′-AAT​CTC​ACA​GCA​GCA​TCT​CGA​CAA​G-3′
	R: 5′-TCC​ACG​GGC​AAG​ACA​TAG​GTA​GC-3′
TNF-α	F: 5′-ATG​GGC​TCC​CTC​TCA​TCA​GTT​CC-3′
	R:5′-CCTCCGCTTGGTGGTTTGCTAC-3′

### 2.8 Immunofluorescence

After the mice were sacrificed and perfused with 4% paraformaldehyde, the lumbar enlargements of the spinal cord were collected and dehydrated with 15 and 30% sucrose. The processed spinal cord specimens were then fixed with an OCT embedding agent and sliced using a Leica CM1860 cryostat. The sections were permeabilized with 0.1% Triton X-100 in phosphate-buffered saline (PBS). After blocking with 5% donkey serum albumin for 1 h, the sections were incubated with the following primary antibodies: mouse anti-VGluT2 antibody (1:200, Abcam, United States), rabbit anti-NeuN (1:200, Abcam, United States), goat anti-Iba-1 (1:200, Wako, Japan), rabbit anti-GFAP (1:200, Abcam, United States), rabbit anti-SYP (1:200, Abcam, United States), rabbit anti-BDNF antibody (1:200, Abcam, United States), and rabbit anti-TrkB (1:200, Abcam, United States) overnight at 4°C. On the next day, the slides were resined in PBS, and then incubated with the corresponding goat, rabbit, or mouse Alexa Fluor 488 or 594-conjugated secondary IgG antibodies (1:200, Jackson ImmunoResearch, United States) in the dark. The slides were rinsed with PBS and mounted with DAPI Fluoromount-G (SouthernBiotech, United States). Images were obtained using a Leica Observer microscope. The images shown in the figures are representative results from three animals per group.

### 2.9 Statistical Analysis

All statistical data are expressed as the mean ± SD. For behavioral tests, groups were compared using two-way repeated ANOVA with post hoc Bonferroni test. For multiple comparisons, one-way ANOVA test was employed, followed by post hoc Bonferroni test. For two group comparisons, unpaired Student’s t-test was employed. Sample sizes were consistent with those reported in similar studies. *p* < 0.05 was considered statistically significant. GraphPad Prism 7.0 was employed for all data analysis.

## 3 Results

### 3.1 VGluT2 Is Upregulated in the Spinal Cord After Chronic Morphine Injections

**FIGURE 1:**
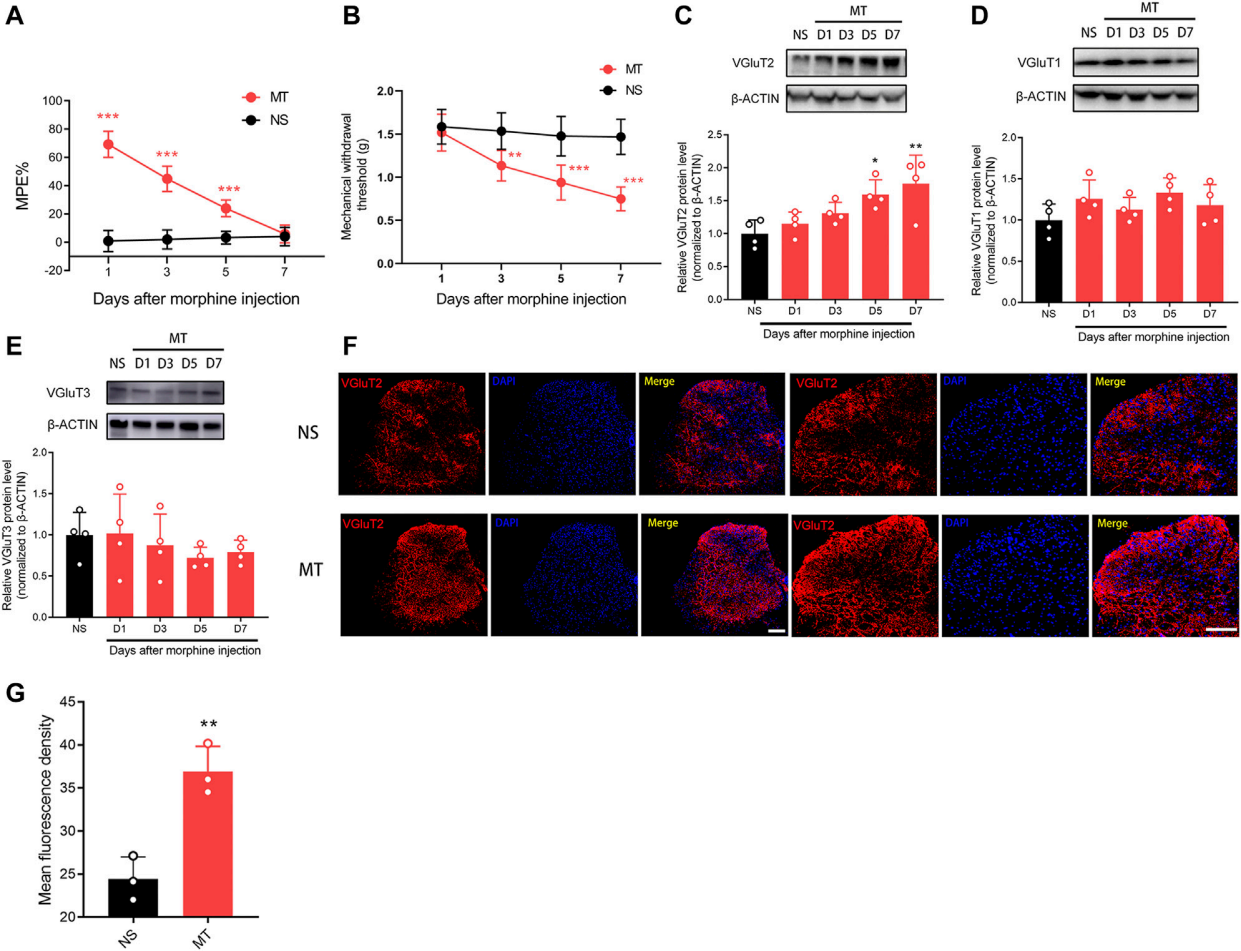
Repeated administration of morphine increases VGluT2 expression in the spinal cord **(A)** Time course of morphine tolerance assessed by the tail-flick test in mice. Tail flick test was performed 30 min before morphine (MT) or saline (NS) injections, 30 min later, the test was performed again. n = 8 mice. MT versus NS, two-way ANOVA, F (1, 14) = 228.2, *p* < 0.001 with Bonferroni correction. ***p < 0.001, compared with NS **(B)** Mechanical pain threshold before morphine or saline injections. n = 8 mice. MT versus NS, two-way ANOVA, F (1, 14) = 37.2, *p* < 0.001 with Bonferroni correction. **p < 0.01, ***p < 0.001, compared with NS **(C-E)** Time course of VGluT1-3 protein level changes in the spinal cord after repeated morphine administration. n = 4, one-way ANOVA **(C)** F (4, 15) = 5.749, *p* = 0.0052 **(D)** F (4, 15) = 1.555, *p* = 0.2372 **(E)** F (4, 15) = 0.6829, *p* = 0.6146 with Bonferroni correction. *p < 0.05, **p < 0.01, compared with NS (D7) **(F,G)** Representative immunofluorescent images **(F)** and quantitative summary **(G)** showing that VGluT2 expression is increased in the lumbar spinal cord after morphine injection on day 7 after morphine administration. Scale bars, 100 μm, n = 3. Student’s unpaired *t*-test, t = 5.550, *p* = 0.0052. **p < 0.01, compared with NS.

Following 7-days of continuous injections of morphine, the %MPE of mice gradually decreased. On day 7, there was no difference in %MPE between the (morphine) MT and (normal saline) NS groups (n = 8, *p* > 0.99; Figure 1A), indicating the successful establishment of morphine tolerance. We found the mechanical pain thresholds of mice also progressively decreased following continuous injections of morphine, but not saline injections, indicating that mice developed mechanical hyperalgesia (Figure 1B). The body weight of mice gained less after chronic morphine administration (Supplementary Figure S1). To identify the expression changes of VGluTs after the chronic administration of morphine, we measured the protein levels of three VGluTs isoforms at different time points. Western blotting results revealed that VGluT2 gradually increased 5–7 days after morphine administration (n = 4, *p* = 0.0052; Figure 1C). Unlike VGluT2, the expression of VGluT1 and VGluT3 remained unchanged after morphine injections (n = 4, *p* = 0.2372; Figure 1D; n = 4, *p* = 0.6146; Figure 1E).

**FIGURE 2:**
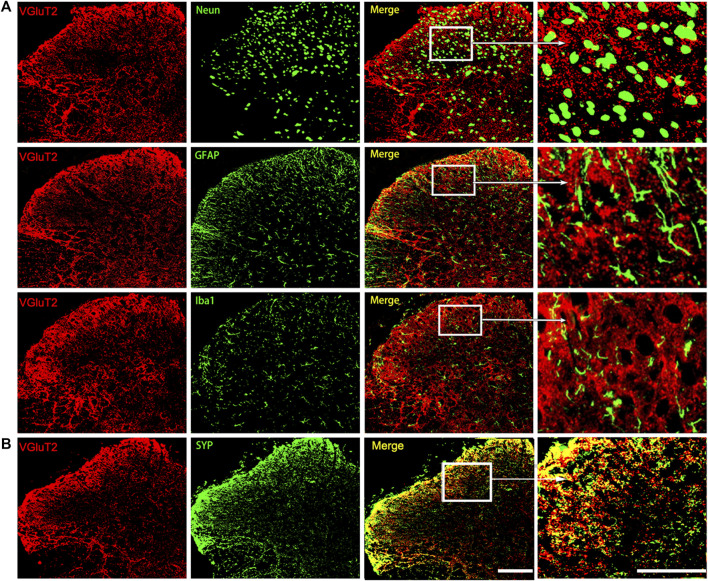
Characterization of the expression profile of VGluT2 in the spinal cord **(A)** Representative immunofluorescence images show VGluT2 is colocalized with neurons (labeled by NeuN), astrocytes (labeled by GFAP), and microglia (labeled by Iba1) in the lumbar spinal dorsal horn from MT mice **(B)** Representative immunofluorescence images show VGluT2 is colocalized with synaptophysin (labeled by SYP) in the spinal dorsal horn from MT mice. n = 4. Scale bars, 100 μm.

We then characterized the spatial expression profile of VGluT2 in the spinal cord. Using immunofluorescence staining, we detected a broad expression pattern of VGluT2 in the spinal cord, which was mainly expressed in the superficial dorsal horn and ventral horn of the spinal cord (Figure 1F). Consistent with the western blotting results, immunofluorescence staining also revealed that VGluT2 is upregulated in the lumbar spinal cord after chronic morphine injections, especially in the dorsal horn of the spinal cord (n = 3, *p* = 0.0052; Figure 1F,G). Double immunofluorescence staining demonstrated that VGluT2 was mainly expressed around neurons (labeled by NeuN), partially co-expressed with either astrocyte (labeled by GFAP) or microglia (labeled by Iba1) (Figure 2A). Furthermore, we evaluated the colocalization profile of VGluT2 and synaptophysin (SYP), a membrane protein of synaptic vesicle which is used as a marker to identify presynaptic axon terminals in the spinal cord. Double labeling demonstrated that VGluT2 was highly co-expressed with SYP, indicating VGluT2 was mainly located in the presynaptic axon terminals (Figure 2B).

### 3.2 Inhibition or Knockdown of Spinal VGluT2 Alleviates the Development of Morphine Tolerance and Mechanical Hyperalgesia

**FIGURE 3:**
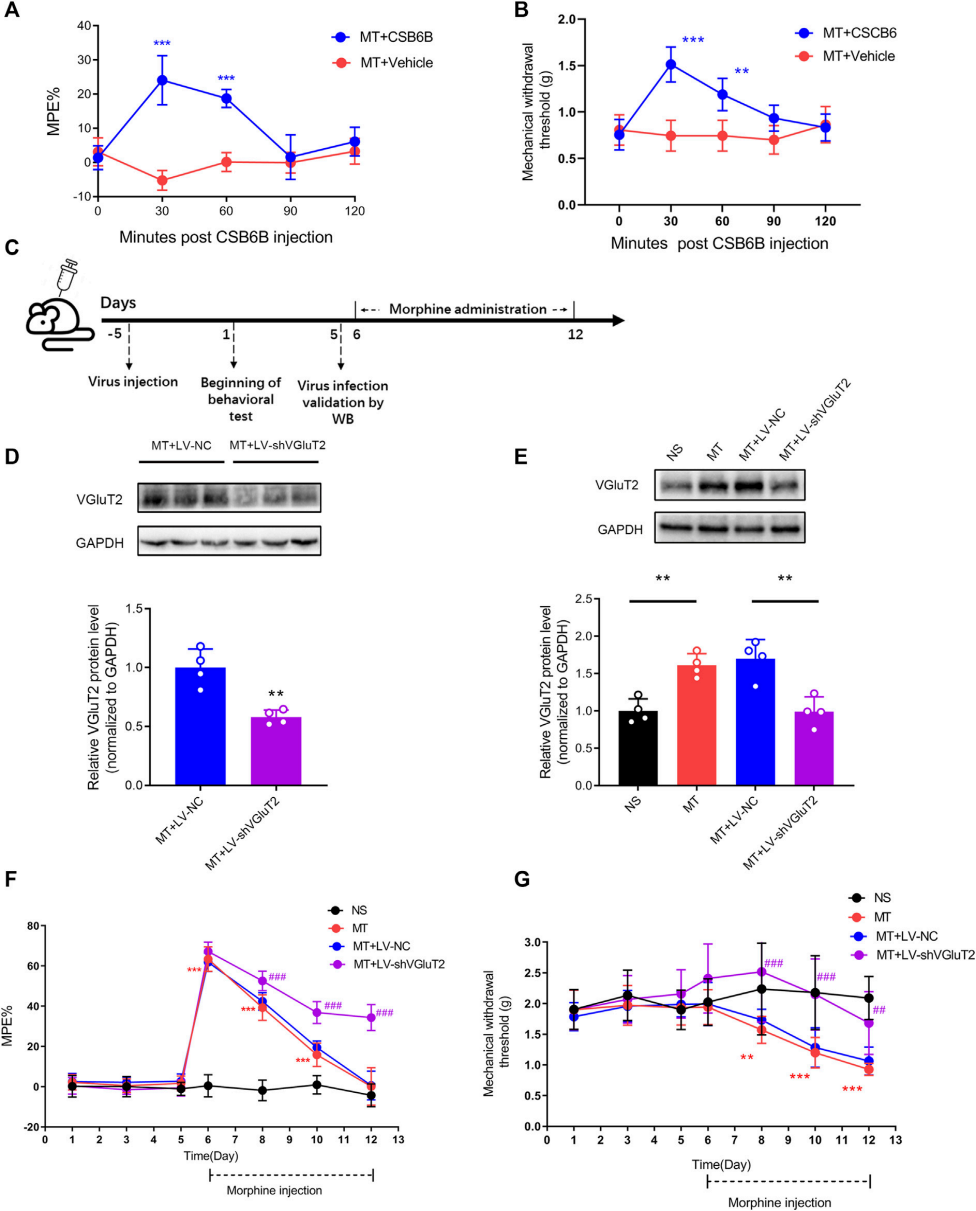
Inhibition or knockdown of VGluT2 attenuates the development of morphine tolerance **(A)** Effect of intrathecal injection of VGluT2 inhibitor CSB6B (5μg/5 μL) on morphine-induced tolerance assessed by the tail-flick test. n = 6, MT + CSB6B versus MT + Vehicle, two-way ANOVA, F (1, 10) = 67.34, *p* < 0.0001 with Bonferroni correction; ***p < 0.001, compared with MT + Vehicle **(B)** Effect of CSB6B (5μg/5 μL) on morphine-induced mechanical hyperalgesia. n = 6, MT + CSB6B versus MT + Vehicle, two-way ANOVA, F (1, 10) = 26.37, *p* = 0.0004 with Bonferroni correction; **p < 0.001, ***p < 0.001, compared with MT + Vehicle **(C)** Experimental schematic diagram showing virus injection, behavioral tests, virus efficiency validation as well as morphine administration in mice expressing scrambled RNA and shVGluT2 **(D,E)** Western blotting test showing efficient VGluT2 knockdown in the spinal cord **(D)** VGluT2 was efficiently knocked down 10 days after intrathecal virus injection (7.5 μL with a titer of 1×10^9^ TU/mL), n = 4. Student’s unpaired *t*-test, t = 4.956, *p* = 0.0026. **p < 0.01, compared with MT + LV-NC **(E)** VGluT2 expression changes after 7-days of consecutive morphine injections. n = 4, one-way ANOVA, F (3, 12) = 14.99, *p* = 0.0002 with Bonferroni correction, **p < 0.01 **(F,G)** Effect of knockdown of VGluT2 on morphine-induced tolerance **(F)** and mechanical hyperalgesia **(G)**. n = 9 mice. Two-way ANOVA **(F)** F (3, 32) = 236.0, *p* < 0.0001 **(G)** F (3, 32) = 15.22, *p* < 0.0001 with Bonferroni correction. **p < 0.01, ***p < 0.001, compared with NS, ^##^ *p* < 0.01, ^###^ *p* < 0.001, compared with MT + LV-NC.

To determine the causal relationship between VGluT2 and the development of morphine tolerance, we first explore the effect of VGluT2 inhibitor CSB6B on morphine-induced tolerance. CSB6B was intrathecally injected immediately after the last morphine injection. 30 min after intrathecal injection of CSB6B, the %MPE of mice was increased compared to those injected with Vehicle (24.08 ± 7.18% versus -5.25 ± 2.90%, n = 6, *p* < 0.001, Figure 3A). The restoration of the antinociceptive effect of morphine lasted for 60 min following CSB6B injection (18.73 ± 2.61% versus 0.13 ± 2.75%, n = 6, *p* < 0.001, Figure 3A). 90 min after the injection, there was no difference between the two groups (n = 6, *p* > 0.99; Figure 3A). The changes in the mechanical thresholds of the mice following intrathecal injection of CSB6B were consistent with the changes in %MPE (Figure 3B). To further investigate how the loss of VGluT2 affects morphine tolerance, we generated a lentivirus expressing shRNA targeting against VGluT2 (Figure 3C). The efficiency of the VGluT2 knockdown was validated by western blotting. 10 days after the LV-shVGluT2 injection, the spinal VGluT2 expression level of mice expressing shVGluT2 significantly decreased compared with mice expressing scrambled shRNA (n = 4, *p* = 0.0026; Figure 3D), and this effect lasted for 17 days after the lentivirus administration (n = 4, *p* = 0.0013; Figure 3E). In the tail-flick test, we found that the knockdown of VGluT2 by lentivirus attenuated the development of morphine tolerance. Beginning on day 3 after morphine injections, the %MPE of mice expressing shRNA was increased compared to mice expressing scrambled shRNA (52.56 ± 4.80% versus 42.38 ± 4.37%, n = 9, *p* = 0.001; Figure 3F). 7 days after morphine administration, mice expressing scrambled shRNA showed no difference in %MPE compared with NS mice (n = 9, *p* = 0.1665; Figure 3F). However, mice injected with LV-shVGluT2 displayed an increase %MPE compared to mice injected with scrambled shRNA (34.40 ± 6.47% versus 0.60 ± 7.08%, n = 9, *p* < 0.001; Figure 3F). Moreover, compared to mice injected with scrambled shRNA, mice injected with LV-shVGluT2 displayed an increased mechanical withdrawal threshold of the hind paw 3 days after morphine injection (2.52 ± 0.82 g versus 1.73 ± 0.17 g, n = 9, *p* = 0.003; Figure 3G), and the alleviation of mechanical hyperalgesia lasted for 7 days after morphine injection (MT + LV-shVGluT2 versus MT + LV-NC, n = 9, *p* = 0.0068; Figure 3G).

### 3.3 Overexpression of VGluT2 Promotes the Development of Morphine Tolerance

**FIGURE 4:**
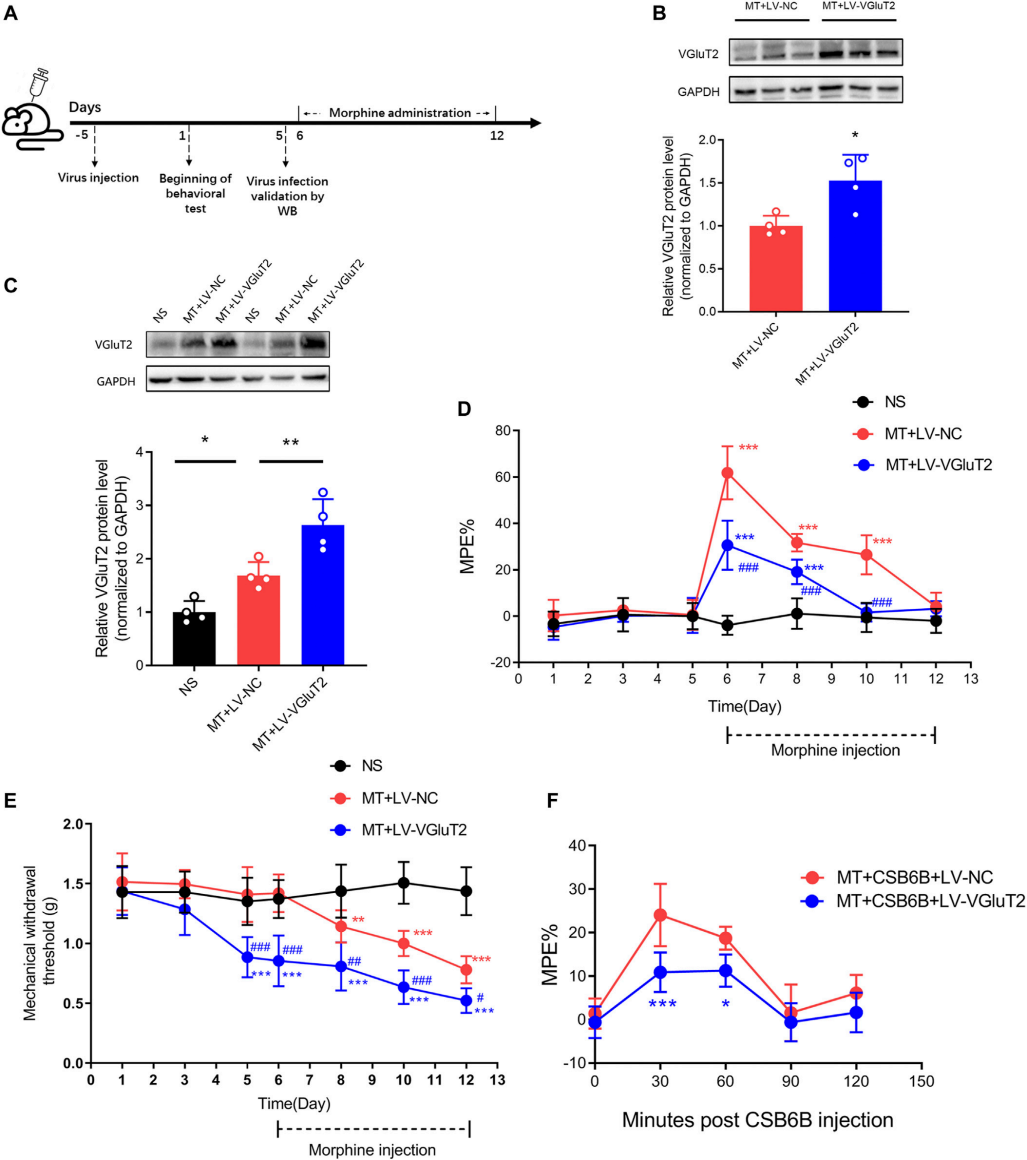
Overexpression of VGluT2 promotes the development of morphine tolerance **(A)** Experimental schematic diagram showing virus injection, behavioral tests, virus efficiency validation as well as morphine administration in mice expressing scrambled RNA and VGluT2 **(B,C)** Western blotting test showing efficient VGluT2 overexpression in the spinal cord **(B)** VGluT2 expression changes 10 days after an intrathecal lentivirus injection (7.5 μL with a titer of 1×10^9^ TU/mL). n = 4, Student’s unpaired *t*-test, t = 3.239, *p* = 0.0177, *p < 0.05, compared with MT + LV-NC **(C)** The expression changes of VGluT2 after consecutive morphine injections on day 12. n = 4, one-way ANOVA, F (2, 9) = 23.55, *p* = 0.0003 with Bonferroni correction; *p < 0.05, **p < 0.01. Western blotting test was performed 10 and 17 days after intrathecal virus injection **(D,E)** Effect of overexpression of VGluT2 on morphine-induced tolerance **(D)** and mechanical hyperalgesia **(E)**. n = 7, two-way ANOVA **(D)** F (2, 18) = 73.66, *p* < 0.0001 **(E)** F (2, 18) = 45.71, *p* < 0.0001 with Bonferroni correction. **p < 0.01, ***p < 0.001, compared with NS, ^#^ *p* < 0.05, ^##^ *p* < 0.01, ^###^ *p* < 0.001, compared with MT + LV-NC **(F)** Effect of overexpression of VGluT2 on CSB6B induced restoration of morphine antinociception. n = 6, two-way ANOVA, F (1, 10) = 16.54, *p* = 0.0023 with Bonferroni correction; *p < 0.05, ***p < 0.001, compared with MT + CSB6B + LV-NC.

To further explore the role of VGluT2 in the development of morphine tolerance, we used a lentivirus expressing VGluT2 to overexpress VGluT2 (Figure 4A). Following western blotting test showed 10 days post lentivirus injection, the spinal VGluT2 expression level in mice injected with LV-VGluT2 was significantly greater than in mice injected with LV-NC (n = 4, *p* = 0.0177; Figure 4B), which confirmed the efficiency of lentivirus infection. This effect was sustained for up to 7 days after morphine administration (n = 4, *p* = 0.0083; Figure 4C). The tail-flick test showed that overexpression of VGluT2 induced a robust decrease in %MPE from day 1 to day 5 after morphine injection compared to mice injected with LV-NC (n = 7, *p* < 0.001; Figure 4D). On day 5 after morphine injection, no significant %MPE was found in mice subjected to LV-VGluT2 or saline injection (1.57 ± 3.92% versus -0.56 ± 6.31%, MT + LV-VGluT2 versus NS, n = 7, *p* = 0.8029; Figure 4D), however, mice injected with LV-NC still had the antinociceptive effect (%MPE = 26.48 ± 8.42%), suggesting overexpression of VGluT2 promoted the formation of morphine tolerance. Moreover, intrathecal injection of LV-VGluT2 decreased the mechanical paw withdrawal threshold and induced mechanical hyperalgesia 10 days after lentivirus injection compared to mice injected with saline (0.89 ± 0.17 g versus 1.35 ± 0.20 g, n = 7, *p* < 0.001; Figure 4E). After morphine consecutive injection, overexpression of VGluT2 exacerbated mechanical hyperalgesia in mice compared with those injected with LV-NC (Figure 4E). Furthermore, we evaluated the effect of LV-VGluT2 on the restoration of morphine antinociception by CSB6B. Tail-flick test showed that 30 min after CSB6B injection, the %MPE of mice expressing LV-VGluT2 was significantly lower than that of mice expressing scrambled RNA (n = 6, *p* < 0.001; Figure 4F), and this effect lasted for 60 min after CSB6B injection (n = 6, *p* = 0.0386; Figure 4F).

### 3.4 Knockdown of VGluT2 Decreased Glutamate Concentration, Suppressed Glial Cells Activation, and Inhibited Pro-inflammatory Cytokines Release

**FIGURE 5:**
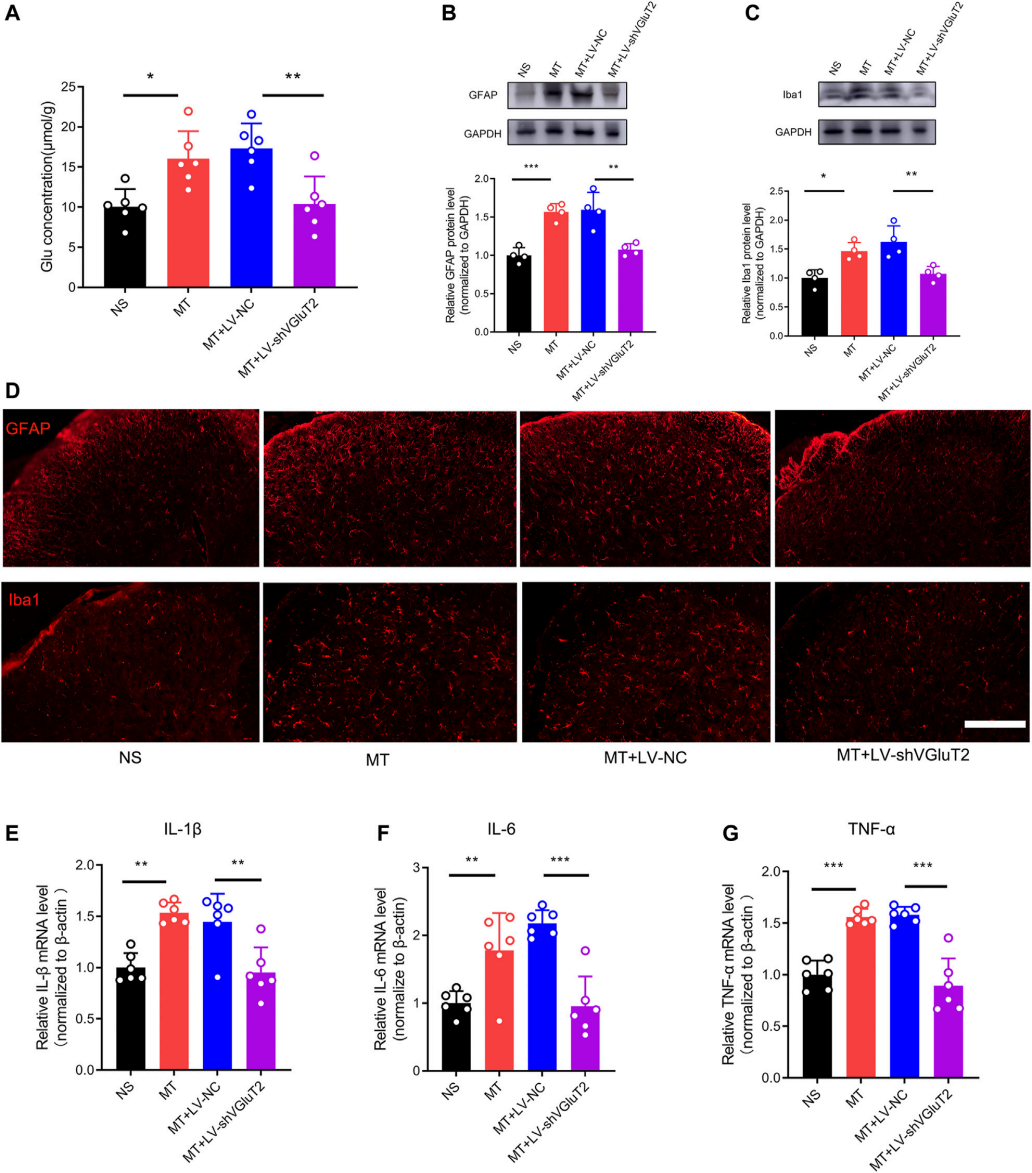
Knockdown of VGluT2 decreases glutamate concentration and suppresses glial cells activation and release of pro-inflammatory cytokines **(A)** Glutamate concentration assay showing knockdown of VGluT2 reduces spinal glutamate concentration. n = 6, one-way ANOVA, F (3, 20) = 8.938, *p* = 0.0006 **(B,C)** Western blotting test showing LV-shVGluT2 decreased GFAP **(B)** and Iba1 **(C)** expression in mice spinal cords. n = 4, One-way ANOVA **(B)** F (3, 12) = 19.97, *p* < 0.0001 **(C)** F (3, 12) = 10.67, *p* = 0.0011, with Bonferroni correction **(D)** Representative immunofluorescent images showing knockdown of VGluT2 reduces morphine-induced upregulation of GFAP and Iba1. n = 4, Scale bars, 100 μm **(E-G)** RT-PCR showing morphine induces-upregulation of pro-inflammatory cytokines were decreased by knockdown of VGluT2. n = 6, One-way ANOVA **(E)** F (3, 20) = 12.97, *p* < 0.0001 **(F)** F (3, 20) = 15.31, *p* < 0.0001 **(G)** F (3, 20) = 31.37, *p* < 0.0001. *p < 0.05, **p < 0.01, ***p < 0.001. The samples used for all tests in this figure are harvested from mice after 7-days repeated morphine administration.

To further explore why the knockdown of VGluT2 contributes to the attenuation of morphine tolerance, we first explored the effect of VGluT2 on spinal glutamate concentration. We found chronic administration of morphine increased spinal glutamate concentration (16.05 ± 3.42 μmol/g versus 10.08 ± 2.18 μmol/g, MT versus NS, n = 6, *p* = 0.015, Figure 5A), and intrathecal injection of VGluT2 shRNA abolished morphine-induced upregulation of glutamate concentration (10.41 ± 3.42 μmol/g versus 17.32 ± 3.13 μmol/g, MT + LV-shVGluT2 versus MT + LV-NC, n = 6, *p* = 0.047, Figure 5A). Then we investigated the effect of dysregulation of VGluT2 on glial cell activation. Western blotting results showed chronic morphine administration increased both GFAP and Iba1 protein expression levels in the spinal cord, and downregulation of VGluT2 by LV-shVGluT2 reduced the upregulation of GFAP and Iba1 (Figure 5B,C). Immunofluorescent staining revealed that chronic morphine administration produced a strong enhancement of astrocytes and microglia expression in comparison with saline-treated mice, especially in the dorsal horn. Pretreatment with lentiviral-mediated shRNA targeting VGluT2 resulted in a significant reduction in astrocyte and microglia expression compared to scrambled RNA-treated mice (Figure 5D). As VGluT2 affects the expression of glial cells, we investigated the expression changes of glial-derived pro-inflammatory cytokines. RT-PCR showed glial-related pro-inflammatory cytokines, including IL-1β, IL-6, and TNF-α were significantly upregulated after chronic morphine administration. Knockdown of VGluT2 diminished morphine-induced upregulation of IL-1β, IL-6, and TNF-α (Figure 5E–G).

### 3.5 BDNF/TrkB Pathway Participates in the Regulation of VGluT2 Expression in Morphine Tolerance

**FIGURE 6:**
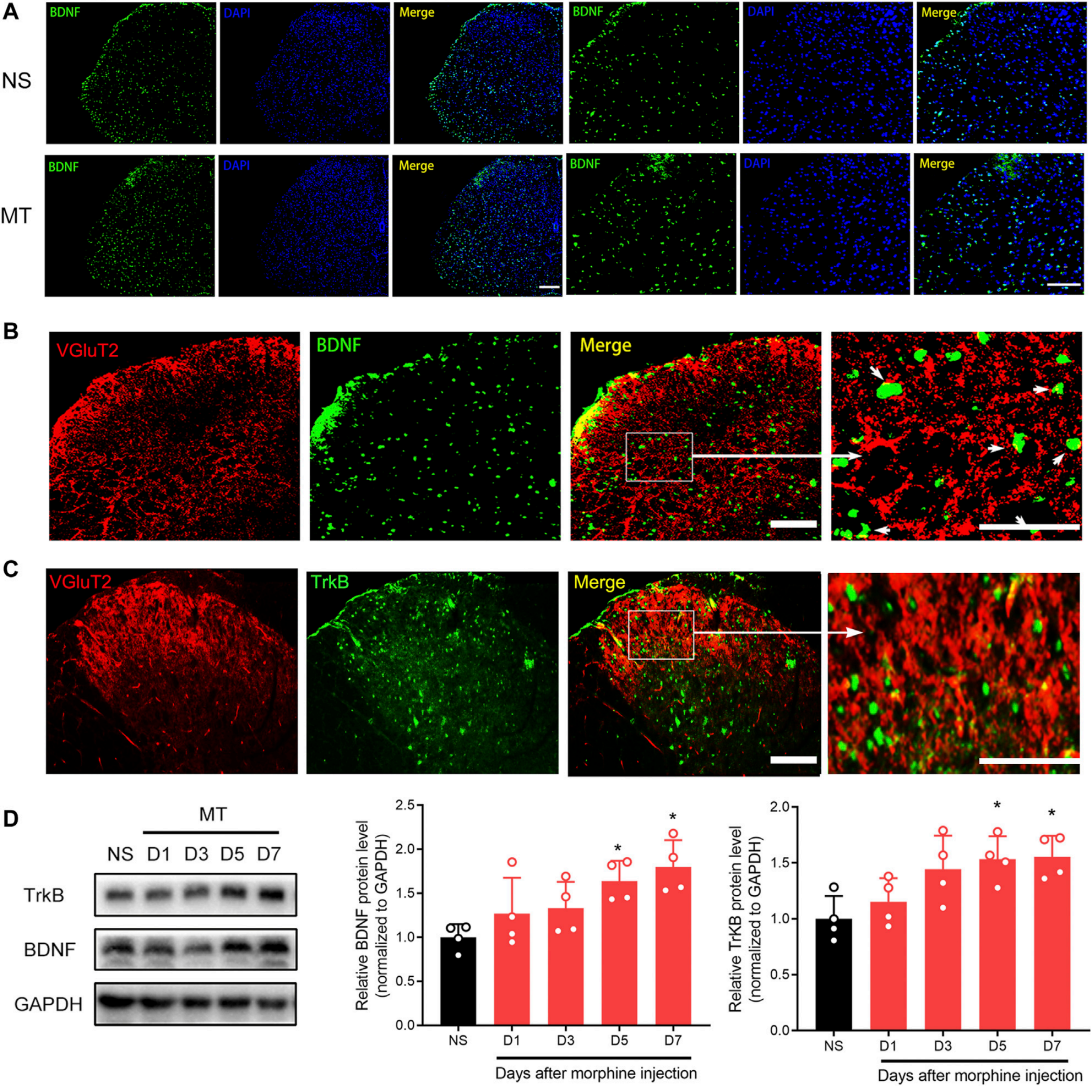
Repeated administration of morphine increases BDNF and TrkB expression in the spinal cord **(A)** Representative immunofluorescent images showing BDNF is increased 7 days after chronic morphine administration. n = 4, Scale bars, 100 μm **(B,C)** Double immunofluorescence staining showing BDNF and TrkB are co-expressed with VGluT2 in the spinal cord of the MT mice. n = 4, Scale bars, 100 μm **(D)** Western blotting showing the time course of BNDF and TrkB in the spinal cord after chronic morphine administration. n = 4, one-way ANOVA (for BNDF) F (4, 15) = 4.669, *p* = 0.0120 (for TrkB) F (4, 15) = 4.808, *p* = 0.0107. *p < 0.05 compared with NS.

**FIGURE 7:**
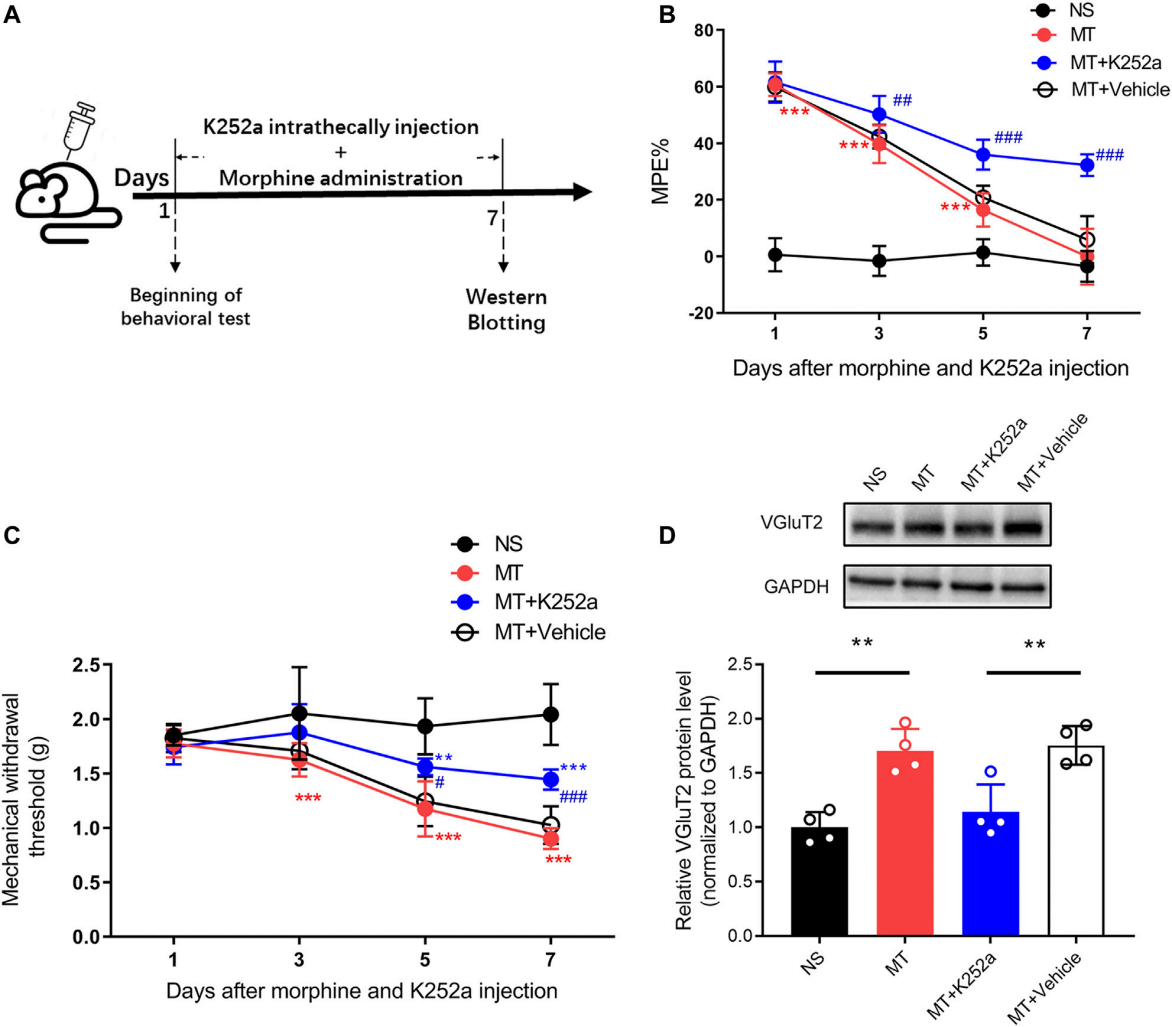
Inhibition of the BDNF/TrkB pathway downregulates the expression of VGluT2 in the spinal cord **(A)** Schematic representation of the experimental procedure **(B,C)** Effect of K252a on morphine induced tolerance **(B)** and mechanical hyperalgesia **(C)**. n = 8, two-way ANOVA **(B)** F (3, 28) = 540.3, *p* < 0.0001 **(C)** F (3, 28) = 38.82, *p* < 0.0001 with Bonferroni correction. **p < 0.01, ***p < 0.001, compared with NS, ^#^ *p* < 0.05, ^##^ *p* < 0.01, ^###^ *p* < 0.001, compared with MT + Vehicle. K252a (0.2 μg/5 μL) was injected intrathecally for seven consecutive days 30 min before each morphine injection **(D)** Western blotting test showing K252a decreases VGluT2 expression in mice spinal cord on day 7 after morphine injections. n = 4, one-way ANOVA, F (3, 12) = 15.14, *p* = 0.0002 with Bonferroni correction. **p < 0.01.

To investigate the effect of the BDNF/TrkB pathway on VGluT2 expression in morphine-tolerant mice, we first explored changes in BDNF expression after chronic morphine administration. Immunofluorescence staining revealed chronic administration of morphine upregulated BDNF expression in the spinal cord (Figure 6A). Double-labeling results demonstrated that VGluT2 was co-expressed with BDNF and TrkB in the dorsal horn of the spinal cord (Figure 6B,C). The following western blotting revealed upregulation of spinal BDNF and TrkB protein levels during the development of morphine tolerance (Figure 6D). To further evaluate the effect of the BDNF/TrkB pathway on the regulation of VGluT2 expression in morphine tolerance, TrkB inhibitor, K252a was employed (Figure 7A). Daily injections of K252a counteracted the occurrence of morphine tolerance and mechanical hyperalgesia. In brief, the decrease in %MPE was prominently restored from day 3–7. On day 7 after repeated morphine injections, the %MPE of mice co-injected with K252a was still higher than mice treated with vehicle (32.33 ± 3.87% versus 5.93 ± 8.31, n = 8, *p* < 0.001; Figure 7B). The effect of K252a on the mechanical pain threshold of the mice was consistent with that of the tail-flick test. On day 3 after repeated morphine injections, the mechanical threshold of mice administered with morphine was lower compared with mice injected with saline (n = 8, *p* < 0.001, Figure 7C). However, mice co-administered with K252a did not develop mechanical hyperalgesia on day 3 (MT + K252a versus MT + vehicle, n = 8, *p* = 0.3329, Figure 7C). The recovery of mechanical hyperalgesia lasted for day 7 after morphine consecutive injections. Western blotting test showed chronic morphine-induced upregulation of VGluT2 expression in the spinal cord was restored by K252a intrathecal injection (Figure 7D).

## 4 Discussion

In our present study, we found chronic morphine administration increased the expression of VGluT2 in the spinal cord. Inhibition or knockdown of VGluT2 in the spinal cord attenuated the development of morphine tolerance and hyperalgesia, while overexpression of VGluT2 facilitated the formation of morphine tolerance. In addition, we found knockdown of VGluT2 decreased spinal glutamate concentration and inhibited morphine-induced activation of glial cells and glial-derived pro-inflammatory cytokines. Moreover, we found BDNF/TrkB pathway was involved in the regulation of VGluT2 expression in morphine tolerance. These findings suggest VGluT2 may represent a promising treatment target for morphine tolerance.

In our present study, we constructed a mouse morphine tolerance model to explore the expression changes of VGluT2 in the spinal cord of mice and to verify its role in the development of morphine tolerance process. We found that VGluT2 was predominantly expressed in the superficial dorsal horn, which is consistent with the previous study (Li et al., 2003). Moreover, for the first time, we found that the expression of spinal VGluT2 was significantly upregulated in a time-dependent manner after consecutive morphine administration. While the expression of the other two VGluT isoforms, VGluT1 and VGluT3, remained unchanged after chronic morphine injection. However, the expression changes of VGluT2 in the spinal cord after the development of morphine tolerance are inconsistent. Suzuki et al. found chronic morphine injections increased VGluT1 expression, while VGluT2 remained unchanged after chronic morphine injection (Suzuki et al., 2006). However, in their study, morphine was administered once a day, whereas, in our study, morphine was administered twice a day. We postulate the difference in drug doses may explain this discrepancy.

Previous studies have demonstrated that selective knockout of VGluT2 can increase the degree of itching in mice, accompanied by a significant decrease in a series of acute and chronic pain (Liu et al., 2010). However, previous studies mainly pay attention to the role of VGluT2 in chronic pain and itching, and few studies have explored the effect of VGluT2 dysregulation on the development of morphine tolerance. In our study, we found intrathecal administration of VGluT inhibitor CSB6B or LV-shVGluT2 suppressed the formation of morphine tolerance and the accompanying hyperalgesia. Subsequently, we overexpressed lentivirus LV-VGluT2 via an intrathecal injection to upregulate the expression of VGluT2 in the spinal cord, which can further promote the development of morphine tolerance and the occurrence of mechanical hyperalgesia. Moreover, we found the overexpression of VGluT2 also abolished CSB6B-induced restoration of the antinociceptive effect of morphine. Taken together, these results suggest VGluT2 plays a pivotal role in the development of morphine tolerance and morphine-induced hyperalgesia.

VGluT2 exerts its biological role by regulating the concentration of intersynaptic glutamate. Its expression level and transport capacity can affect the rate and degree of vesicle filling with glutamate and determine whether the vesicle releases glutamate or not (Daniels et al., 2006; Herman et al., 2014). Thus we evaluated the effect of dysregulation of VGluT2 on glutamate concentration in the development of morphine tolerance. We found that the spinal concentration of glutamate was significantly increased after continuous morphine injection, which is consistent with previous reports (Wen et al., 2004). In addition, downregulation of VGluT2 by shRNA reduced the release of glutamate in the spinal cord. As glutamate plays an important role in the formation and development of morphine tolerance, this result could, at least in part, explain our results regarding the ability of VGluT2 to regulate morphine-induced tolerance.

It has been reported that chronic morphine injections can induce spinal glial cell activation (Song and Zhao, 2001; Harada et al., 2013). The activation of glial cells promotes the release of pro-inflammatory cytokines, which further promotes neurotransmitters release and increases the excitability of nearby neurons, and finally induces central sensitization (Raghavendra et al., 2002). Moreover, increased glial-derived cytokine induces the activation of astrocytes and microglia, resulting in the further release of pro-inflammatory cytokines, which oppose the analgesic actions of morphine (Eidson and Murphy, 2019). Our previous study has demonstrated that dysregulation of VGluT2 is correlated with the activation of spinal glial cells during the development of chronic pain (Wang et al., 2019). In our present study, we found increased spinal VGluT2 expression was paralleled by the activation of glial cells and upregulation of pro-inflammatory cytokines after chronic morphine administration. However, the knockdown of VGluT2 inhibited the activation of glial cells. In addition, the upregulation of the pro-inflammatory cytokines, IL-1β, IL-6, and TNF-α, were also reduced after LV-shVGluT2 injection. These results suggest VGluT2 modulates the development of morphine tolerance possibly via regulating the activation of spinal glial cells and pro-inflammatory cytokines release.

BDNF is an important member of the neurotrophic factor family, which exerts an important role in the formation and development of morphine tolerance. Previous studies have reported that continuous morphine administration increases BDNF expression in the spinal cord of mice, and inhibition of BDNF expression delays the occurrence of morphine tolerance (Matsushita et al., 2013; Rozisky et al., 2013; Sikandar et al., 2018). Another study reported that blocking BDNF/TrkB pathway restored spinal KCC2 (K^+^- Cl^−^ co-transporter) function, preserved Cl^−^ homeostasis in the spinal cord, and reversed morphine-induced hyperalgesia (Ferrini et al., 2013). The combination of BDNF and TrkB can further activate downstream signaling pathways, then activate CaMKII and other kinases, and finally, activate CREB and other transcription factors to regulate the expression of related proteins (Sasi et al., 2017). Melo et al. found that in hippocampal neurons *in vitro*, the expression levels of VGluT2 were significantly upregulated after exogenous BDNF stimulation, while TrkB inhibitors inhibited the upregulated expression of VGluT2 induced by BDNF, indicating that the BDNF/TrkB pathway participates in regulating the expression of VGluT2 (Melo et al., 2013). Another study by Azogu et al. found that the injection of TrkB antagonist ANA-12 in the nucleus accumbens could reduce anxious behaviors in stressed rats and lead to the downregulation of VGluT2 expression in the hypothalamus and amygdala (Azogu and Plamondon, 2017). However, the role of the BDNF/TrkB pathway in the regulation of VGluT2 in the spinal cord has not yet been studied. In this study, we found that the expression of BDNF and TrkB in the spinal cord of morphine-tolerant mice was significantly upregulated. We also found for the first time that there is a large amount of colocalization of BDNF and VGluT2 in the spinal dorsal horn, and colocalization mainly occurs in the superficial dorsal horn related to pain, which suggests that BDNF may be spatially related to VGluT2. Moreover, intrathecal administration of the BDNF/TrkB pathway-specific inhibitor K252a downregulated the expression of VGluT2 in the spinal cord and delayed the occurrence of morphine tolerance and hyperalgesia in mice. These results further suggest that the expression of VGluT2 in the spinal cord of morphine tolerant mice may be regulated by the BDNF/TrkB signaling pathway.

Based on our findings, we propose a possible mechanism for the regulation of VGluT2 expression by BDNF/TrkB that participates in morphine tolerance. Continuous application of morphine upregulates BDNF expression in the central nervous system. BDNF acts on the TrkB receptor on the cell surface to further activate its downstream signaling pathways and transcription factors, promotes the transcription of VGluT2, and increases its expression. The upregulated VGluT2 promotes the entry of more glutamate into the synaptic vesicle and further promotes the release of glutamate in the synaptic vesicle, which significantly increases glutamate level in the synaptic cleft, activates astrocytes and microglia, promotes the release of pro-inflammatory cytokines, induces central sensitization in the spinal cord, and finally promotes the formation and development of morphine tolerance. This study expands our understanding of the function of VGluT2 in the spinal cord and provides new therapeutic targets and strategies for the prevention and treatment of morphine tolerance.

## Data Availability

The raw data supporting the conclusions of this article will be made available by the authors, without undue reservation.
