# Supplementary material for: Dysregulation of Vesicular Glutamate Transporter VGluT2 via BDNF/TrkB Pathway Contributes to Morphine Tolerance in Mice
Source: Front Pharmacol. 2022 Apr 26;13:861786. doi: 10.3389/fphar.2022.861786 (PMC9086316; doi:10.3389/fphar.2022.861786)
Supplement: Supplementary file 1 [file Image1.pdf]

Supplementary Material

Supplementary Figures

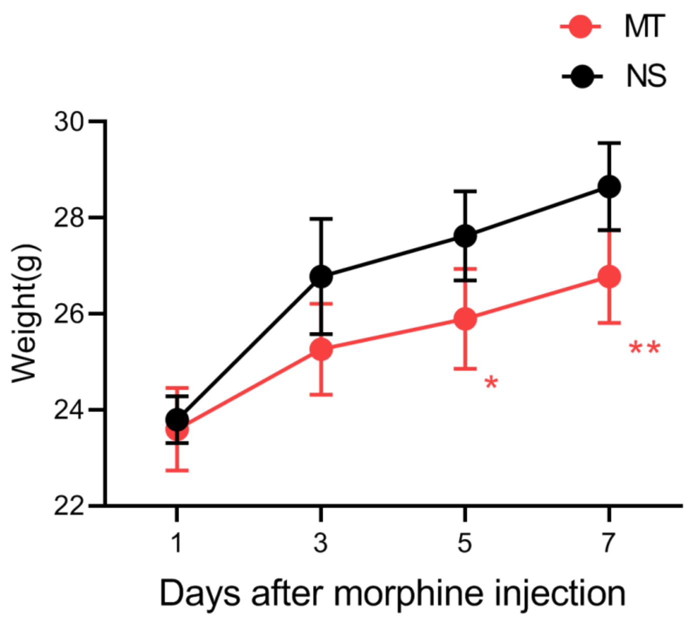

Supplementary Figure 1. The change of body weight after morphine or saline injections. n=8 mice. MT versus NS, two-way ANOVA, $F (1, 14) = 21.4$, $P<0.001$ with Bonferroni correction. *$P<0.05$, **$P<0.01$, compared with NS.
